# Editorial: Digital twins in medicine—transition from theoretical concept to tool used in everyday care

**DOI:** 10.3389/fdgth.2025.1573727

**Published:** 2025-02-27

**Authors:** Stephen Gilbert, David Drummond, Fabienne Cotte, Tjalf Ziemssen

**Affiliations:** ^1^ Else Kröner Fresenius Center for Digital Health, TUD Dresden University of Technology, Dresden, Germany; ^2^ Department of Pediatric Pulmonology and Allergology, University Hospital Necker-Enfants Malades, Paris, France; ^3^ Department of Emergency Medicine, University Clinic Marburg, Philipps-University, Marburg, Germany; ^4^ Center for Clinical Neuroscience, Carl Gustav Carus University Hospital, Dresden, Germany

**Keywords:** digital twin, clinical decision support, artificial intelligence, prediction model, personalised medicine, precision medicine

**Editorial on the Research Topic**
Digital twins in medicine—transition from theoretical concept to tool used in everyday care

This Research Topic gathers different contributions addressing the practical advancement of the concept of digital twins in medicine, moving it form a vague theoretical concept towards the foundation to tools used in everyday healthcare. The digital twin (sometimes known as a virtual twin) is a concept that is mainstream in manufacturing, where a digital representation is created of an intended or actual real-world physical product, system, or process (the physical twin). The digital twin serves as an effectively indistinguishable digital counterpart to the physical twin and is used for practical purposes such as simulation, monitoring and maintenance (1). This concept has existed in medicine for decades, but unlike in industry, it has not found its way to practical day-to-day application in patient care (2, 3). Despite this there is renewed research interest in this theme.

The goal of this Research Topic was to address if we are at the dawn of the digital twin in medical practice and to explore what is needed to realize this. The articles help to define the aspects of digital twin research that are near to translation and those that need substantially more preclinical development before practical application is possible. Digital Twins of patients, which have been defined in various ways such as “a viewable digital replica of a patient, organ, or biological system that contains multidimensional, patient-specific information and informs decisions” (4), involve not only new forms of representation of information about patients, but also simulation methods and often AI-based predictive analytical methods. There is much hype and excitation about medical AI, but medical AI will only delivery its promise if firmly linked to the status of the patient in data—in other words to the digital twin. These raise regulatory and ethical questions, with differing approaches in differing countries—a goal of the Research Topic was to bring some clarity to these challenges alongside different proposed strategies and developments, and as such to serve as a description of the state of the art and path to impact of digital twins in medicine.

The first article of this Topic (Laubenbacher et al.) explores, through an integrative review, the challenge of how personalized medicine can possibly capture enough of the complexity that accompanies each individual patient to determine an optimal way to keep them healthy or restore their health. If the personal patient parameters relevant to disease progression are not considered in decision support model outputs, then personalised medicine will remain an unreaslised dream. Digital Twins therefore require sufficient resolution, i.e., enough mechanistic information to enable them to provide actionable information to the clinician. This article acknowledged that Digital Twin technology for health applications is still in its infancy, and that extensive further research and development is required before we see their impact. This review article focuses on several projects in different stages of development that are anticipated to lead to specific and practical medical Digital Twins or Digital Twin modeling platforms, for example the multiple sclerosis digital twin (5). The article emerged from a 2-day forum on problems related to medical Digital Twins, with a focus on those modelling immune system components. The article also includes highly relevant open access video recordings of the forum discussions.

The second article (Grieb et al.) describes methods for digital twin model for evidence-based clinical decision support in multiple myeloma treatment, including a description of the model architecture, and with a focus on explainability and interpretability in treatment evaluation. Multiple myeloma is a disease that is particularly suited to the application of digital twin approaches. There has been substantial progress in the treatment landscape over the last decades, but despite the efficacy of new medications, patient responses remain highly unpredictable and challenging to address by clinicians and the need for data-driven decision support tools is clear, with some already in use. The authors describe a similarity-based digital twin approach that matches patients with similar historical cases to predict treatment outcomes. Requirements were defined from scientific and technical literature and a four-layer approach was implemented. The digital twin suggests multi-line treatment strategies with the integration of external evidence with transparency in the data processing logic. The article sets out an initial approach to clinical evaluation and illustrates the approach through a detailed description of an exemplary use case for multiple myeloma.

The third article of this Research Topic (Zhang et al.) is an original article that describes a digital twin framework for type 2 diabetes that integrates machine learning with multiomic data, alongside both knowledge graphs and mechanistic models. The researchers developed predictive machine learning models to forecast disease progression using a substantial dataset comprising clinical measurements and multiomic profiles. Knowledge graphs were employed to interpret and provide context to disease relationships. Promise is demonstrated through the modeling framework reaffirming known targetable disease mechanisms and features. The approach has potential as a DT system for precision medicine.

The final article of this Research Topic is a mini review of the role for digital patient twins for personalized therapeutics and pharmaceutical manufacturing (Fischer et al.). The authors set out how digital twins of patients pave the way, not only for decision support systems and improved disease monitoring (as described in the previous three articles) but also facilitate the development of personalized therapeutics through their approach to the management, analysis, and interpretation of medical data. The authors identify some gaps that need to be filled before this can be part of routine as reliable simulations for the prediction of individual drug responses are only available for a minority of scenarios. The article describes how patient digital twin led individualized pharmaceutical manufacturing could function and describes challenges in automation, scalability, the control of costs and limitations in current regulatory steps.

Are digital twins in medicine ready for the real time, i.e., ready to transition from a theoretical concept to a tool used in everyday care. All articles in this Research Topic acknowledge that DT models still face significant challenges in development, including availability of clinical data to algorithmically derive approaches for clinical decision support. They also face challenges in delivering trustworthy decision support to clinicians. A recent systematic review identified 80 unique digital twins being developed for clinical use, of which 98% were still in preclinical phases, further confirming that while the field is rapidly evolving, significant validation work remains needed before widespread clinical adoption (4). Together the articles of this Research Theme propose approaches that mitigate the regulatory and ethical challenges and provide a roadmap for broad clinical adoption. We are still in the nascency of digital twins, as regulatory approved tools, at least in the broader context of the definition of a digital twin, i.e., virtual representations of a patient's health and disease, facilitating real-time diagnostic and therapeutic decision support, prediction of disease progression, monitoring, optimization of care delivery, precision therapeutic manufacture, and ultimately, the use of these approaches to holistically deliver better outcomes. Not all approaches described are immediately ready for translation and not all interoperability and standardization challenges have been solved. There remain challenges in the readiness of DT architectures for regulatory, legal and ethical frameworks, and, as well as challenges in the regulatory frameworks' suitability for digital twins (3, 6). All approaches described require further clinical validation and health technology assessment before they can receive full approval, achieve widespread adoption, and qualify for reimbursement. This Research Topic has helped set out the roadmap for the further development of this promising medical approach.
